# A video-oculography study of fixation instability in myasthenia gravis

**DOI:** 10.3389/fneur.2025.1493418

**Published:** 2025-02-21

**Authors:** Elle Minh Ngoc Le Nguyen, Meaghan J. Clough, Joanne Fielding, Owen B. White

**Affiliations:** ^1^Department of Neuroscience, Monash University, Melbourne, VIC, Australia; ^2^Alfred Health, Melbourne, VIC, Australia

**Keywords:** myasthenia, video-oculography, fixation instability, neuromuscular junction, ocular myasthenia

## Abstract

**Introduction:**

Myasthenia gravis (MG) is an autoimmune disease that causes extraocular muscle weakness in up to 70–85% of patients, which can impact quality of life. Current diagnostic measures are not very sensitive for ocular MG. This study aimed to compare fixation instability (inability to maintain gaze on a target) in patients with MG with control participants using video-oculography.

**Methods:**

A prospective study of 20 age-and sex-matched MG and control participants was performed using a novel protocol with the EyeLink 1000 plus ©. Bivariate contour ellipse area (BCEA) analysis, number of fixations on a target, and percentage of dwell time of fixations in the target interest area (IA) were calculated. Inter-eye (right vs. left) comparisons were performed using paired *t*-tests, and inter-group (MG vs. control) comparisons were performed using independent samples *t*-tests.

**Results:**

There were no inter-eye differences in the BCEAs between control eyes and MG eyes. However, the BCEAs were larger in both the right (RE) and left (LE) eyes of MG patients in the right (RE *p* = 0.029, LE *p* = 0.033), left (RE *p* = 0.006, LE *p* = 0.004), upward (RE *p* = 0.009, LE *p* = 0.018), and downward (RE *p* = 0.006, LE *p* = 0.006) gaze holds of the controls. The total mean sum of gaze hold fixations in all directions was greater in MG patients than in control participants (354 ± 139 vs. 249 ± 135, *p* = 0.020), with horizontal gaze holds showing greater differences than vertical gaze holds (*p* = 0.007 vs. *p* = 0.097). The percentage of dwell time in the target IA was lower in MG patients, but this only reached significance in the right gaze hold (*p* = 0.003).

**Conclusion:**

MG patients showed greater BCEA values and refixations and lower target IA percentages of dwell time during gaze hold than control participants, suggesting extraocular neuromuscular junction instability and fatigue. Interestingly, there were no significant inter-eye differences in MG participants. This study is limited by the small number of patients but adds to the current literature exploring video-oculography in MG patients as a novel diagnostic tool. Further studies are recommended for translation into clinical practice.

## Introduction

Myasthenia gravis (MG) is a phenotypically variable autoimmune disease of striated muscle that can cause disabling ocular symptoms (diplopia, blurred vision, and ptosis) and can progress to more generalised symptoms, such as bulbar, respiratory, and skeletal muscle dysfunction. Several studies, using various eye-tracking methods, have been carried out to identify unique eye movement profiles of MG patients (1, 2); however, no study at this stage has been translated into modern clinical practice. Current standard diagnostic methods lack sensitivity in detecting patients with ocular MG without ptosis (e.g., serology, repetitive nerve conduction, ice test, and Cogan’s lid twitch test), require highly specialised training (e.g., single-fibre EMG), or may cause systemic side effects (e.g., edrophonium). Therefore, there is still a need to find more sensitive and less invasive methods of diagnosis.

Fixation instability has been previously observed on clinical examination of MG in previous studies (33, 34). Bivariate contour ellipse area (BCEA) analysis (3) uses oculography to quantify fixation instability and has been studied in other ophthalmic conditions (4–7). BCEA is an elliptical area that contains fixation points for a given proportion (P) of eye positions during a fixation trial. A smaller BCEA value indicates less spread of fixations and thus more stable fixation, whereas a larger BCEA value indicates more spread of fixations, thus demonstrating instability. One study with 10 myasthenia patients in a Japanese population found that MG patients exhibited upward gaze instability compared to controls (8). Our study aimed to use BCEA with a differing methodology, using a larger number of patients and other gaze directions, to further explore BCEA as a method to identify fixation instability in MG patients compared to control participants.

## Materials and methods

### Standard protocol approvals, registration, and patient consent

The Alfred Health Human Research and Ethics Committee (Project ID 577/19) approved this study, and all participants provided written informed consent.

### Study design and testing

#### Study population

A total of 20 MG patients (formally diagnosed by a neurologist based on paraclinical and clinical testing) were consented and recruited from the outpatient clinics, and 20 age-and sex-matched controls were consented and recruited from the community.

#### Study criteria

The inclusion criteria for MG patients were as follows: (1) age greater than 18 years old; (2) confirmed diagnosis of MG by serology, electrophysiology, or a positive ice test; and (3) visual acuity equal to or better than 6/6 corrected and no visual field defects. The exclusion criteria were as follows: (1) the presence of other ocular motility disorders or central nervous system disorders and (2) a communication or language barrier. Control participants were >18 years of age, did not have a diagnosis of MG or any systemic disease affecting eye movements, and had the same exclusion criteria as the MG patients.

#### Video-oculography (VOG) testing

**Figure 1:**
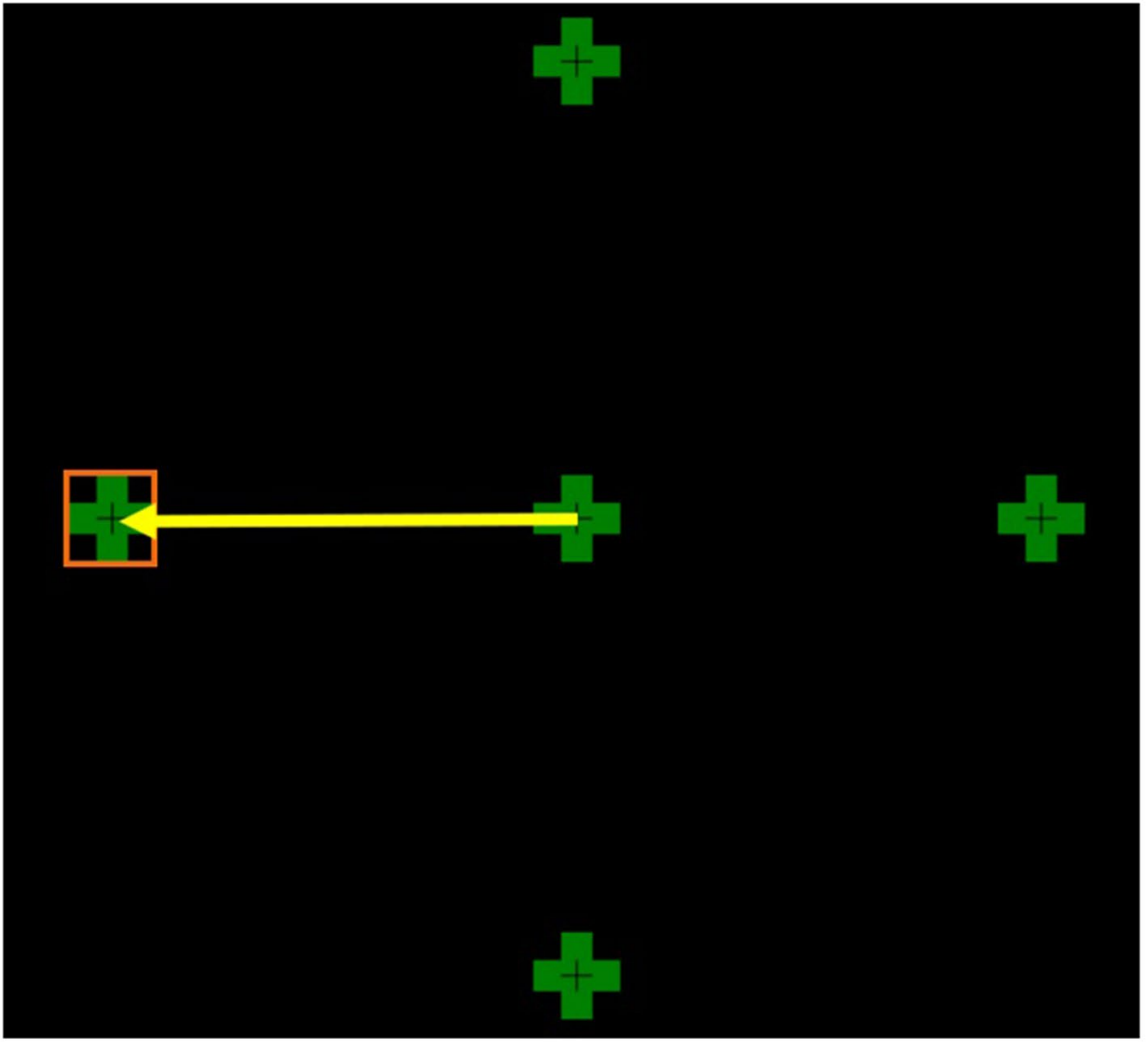
This figure shows all four eccentric targets at 8 deg from the centre. The yellow arrow represents a saccade to the left target. The orange square depicts the left target interest area.

Participants were tested in the morning before 12 pm, and MG patients were asked to continue their medication to prevent exacerbations and significant fatigue during testing. Before testing, participants had a 2-min rest period in the dark with their eyes closed, and a 2-min rest between each trial. Binocular eccentric gaze hold (GH) was recorded using an EyeLink 1000 plus tracker desktop mount, with the pupil position as the eye-tracking principal target. It has a resolution of 0.01 deg and a binocular sampling rate of 1,000 Hz. A chin rest was used to stabilise the heads of the participants. A moving green cross (1.5 degrees (deg) wide and 1.5 deg high), with a smaller, darker cross at the centre was used as a target on an LCD screen (1,920 × 1,080 resolution, 546 mmW, 306 mmH) (Figure 1). The screen was placed 950 mm away from the participant’s line of sight. Each participant’s eye movements were calibrated prior to testing and recalibrated where required, and drift correction was applied during testing.

Each participant had four trial directions in which the green cross moved 8 deg from the centre to an eccentric position (left, right, up, or down) and maintained gaze hold for 30 s. Eight degrees was chosen as it closely represents physiological eye movements. Larger saccades >15 deg were found to lead to inaccurate eye movements.

Trial directions were randomised and counterbalanced for each participant. Participants were advised to try not to blink, if possible, during the 30-s gaze hold (GH) period on the target.

### Data collection and statistical analysis

For each participant, the GH fixation trials were manually visualised before data extraction to ensure the accuracy of the data. A target interest area (IA) encompassing the width and height of each target cross, totalling an area of 2.25 deg^2^, was coded for refixation and BCEA analysis. The IAs act as a reference point when analysing the data (see Table 1).

**Table 1:** Interest area borders (in pixels), for each eccentric target.

Target IA (8,100 pixels^2^)	Left border	Right border	Top border	Bottom border
Left	435	525	495	585
Right	1,390	1,480	495	585
Upward	910	1,000	20	110
Downward	910	1,000	970	1,060

**Figure 2:**
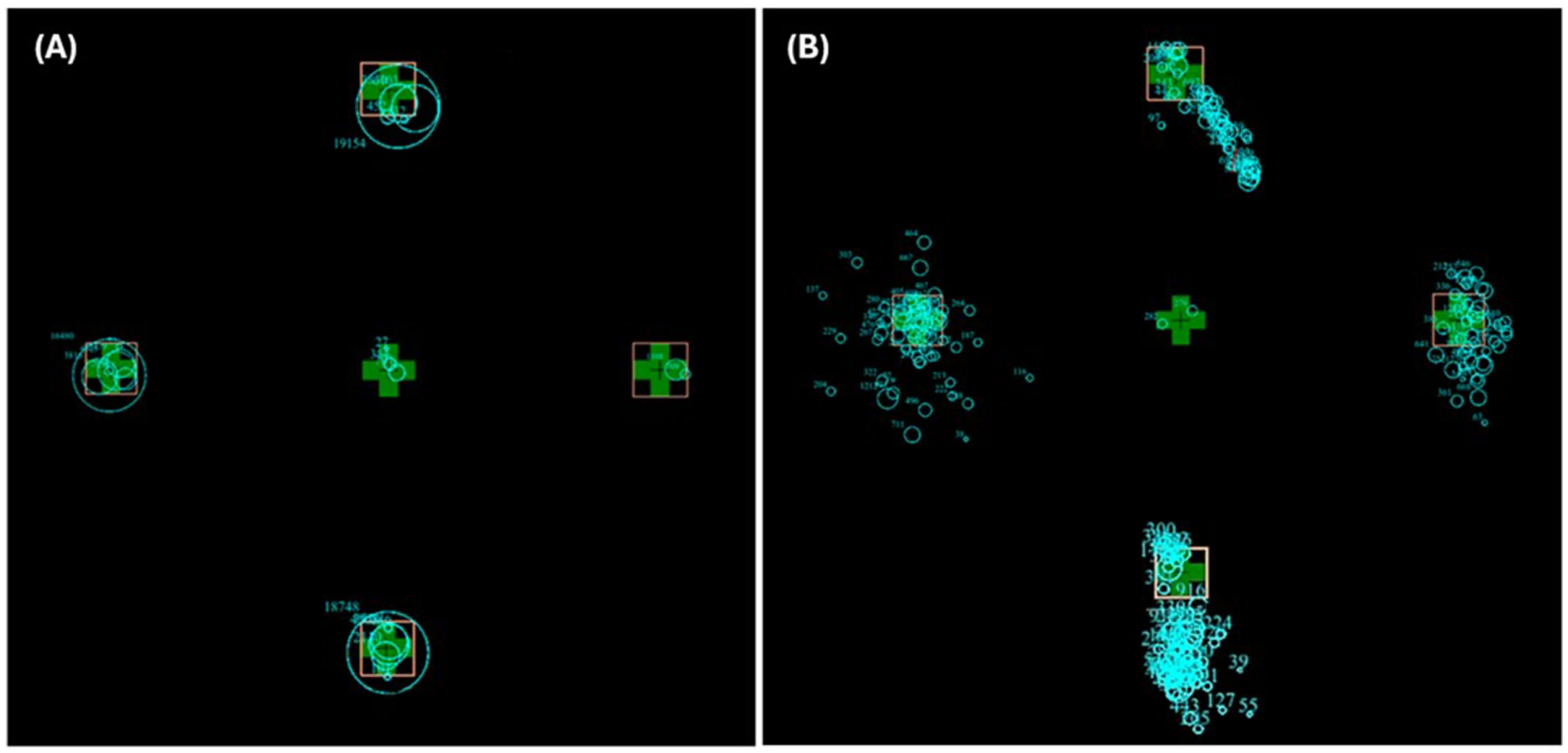
Superimposed raw trial images of a typical control participant **(A)** and MG patient **(B)** in all four trial directions. The blue circles represent fixations, and the size of the circle represents time spent fixating at that location.

For refixation analysis (looking at microsaccades), the number of fixations was counted during the 30-s gaze hold after the initial saccade from the centre (not including any centre fixations). The target IA percentage of dwell time was calculated by dividing the time fixations spent within a target interest area (in ms) by 30,000 ms and multiplying by 100. (The raw data of a typical control and MG patient during a 30-s gaze hold are in Figure 2).

For BCEA analysis, any erroneous pupil capture and blink sequences were removed before extraction. In contrast to the study by Mihara et al. (8), we used data from both eyes, not just the dominant eye, to look at conjugacy (the difference between a participant’s right and left eyes). After cleaning up the raw data, the standard deviation data of the X-and Y-axis pixel coordinates were extracted from each trial direction for each participant (*N* = 320 sets of X–Y coordinates, total of both right and left eyes). Pixel coordinates were converted to degrees of visual angles for analysis.

All analyses were conducted using the statistical software SPSS(c) v29. The mean and standard deviation (SD) for each set of fixation X and Y coordinates were also calculated. From this, Pearson’s moment correlation coefficients (*ρ*) were calculated for each set of X and Y coordinates.

To calculate the BCEA, the following formula was used (3):


$$\mathrm{BCEA=2k}\pi \sigma_{H}\sigma_{V}{\left(1-p^{2}\right)}^{\frac{1}{2}}$$

Using a probability factor of *p* = 0.95 (BCEA encompassing 95% of fixations) and k = 2.99, calculated from the formula *p* = 1 − e^−k^. σ_H_ represents the SD of the X-axis values, and σ_V_ represents the SD of the Y-axis values. *ρ* = Pearson’s moment correlation coefficient for each X-Y standard deviation for each eye. A total of 160 BCEA values were calculated for analysis (20 MG and 20 controls in 4 gaze directions).

Paired *t*-tests were conducted to look at the conjugacy of the right and left eyes (inter-eye differences) of the controls and MG patients separately. Independent samples *t*-tests were conducted to compare eyes between the controls and MG patients (inter-group differences).

## Results

### Demographics

Participant demographics are summarised in Table 2. There were no statistically significant differences in age (*p* = 0.070), sex (*p* = 0.890), ethnicity (*p* = 0.815), refractive error (*p* = 0.349), or hours of sleep (*p* = 0.668) between the control and MG participants. There were no reports of general fatigue or somnolence at the commencement of testing. However, 25% of patients reported feeling fatigued during the test. In total, 45% of MG patients had intermittent complaints of diplopia on a day-to-day basis before testing, but during clinical examination, only 25% of patients reported diplopia on prolonged lateral gaze. During VOG testing, 25% of patients reported diplopia, but they were not the same patients who had reported diplopia during clinical examination.

**Table 2:** Demographics of control and myasthenia gravis (MG) participants.

	Control *N* = 20	MG *N* = 20
Age (mean years, SD)	45.2 ± 15.6	53.5 ± 14.53
Female subjects (*N*)	11	12
Ethnicity (*N*)		
Caucasian	9	13
Asian	10	6
Other	1	1
Refractive error (*N*)^*^		
Nil	9	11
Myopia	8	6
Hypermetropia	3	3
Hours of sleep	7.03 ± 0.97	6.79 ± 2.01
EOM (*N*)		
Horizontal restriction	0	1
Vertical restriction	0	2
Phoria	0	0
Tropia	0	1
Ptosis (*N*)	0	2
Diplopia on 30 s beside lateral GH (*N*)	0	5
MGCS (score out of 50), (*N*)		
0	NA	9
1–10		9
10–20		2
21–50		0
Current treatment (*N*)		
Pyridostigmine		10
Prednisolone		5
Mycophenolate mofetil		5
Azathioprine		2
IVIg		1
PLEX		0
Rituximab		0
Thymectomy		1

* Corrected during testing.

ACh-R, acetylcholine receptor; EOM, extraocular eye movements; IVIg, intravenous immunoglobulin; GH, gaze hold; MuSK, muscle-specific kinase; MGCS, myasthenia gravis composite score; PLEX, plasma exchange; SFEMG, single-fibre electromyography.

A total of 65% of MG patients were purely ocular (only had eye symptoms and signs), and the rest were generalised MG (also affecting other parts of the body). Regarding diagnostic testing, 65% of MG patients had a positive anti-AChR-Ab, one had a positive MuSK Ab, and 20% of MG patients were diagnosed via SFEMG. One patient was diagnosed previously using edrophonium testing, and one had a clinically positive ice test. The average duration from diagnosis was 6.06 ± 8.41 years. The majority of MG patients were under medical treatment at the time of testing.

### Initial saccades to eccentric target GH

Although not the focus of this study, 160 initial saccades to the target (20 saccades × 4 trial directions × 2 participant groups) were visualised manually. The percentage of hypometric saccades was 31.6% in controls, compared to 51.3% in MG patients. Hypermetric saccades were observed in 25.3% of control participants and 10.3% of MG patients. Only one control and one MG patient had a quiver in this cohort. A total of 10.1% of control participants exhibited backdrifts compared to 16.2% of MG patients. It should be noted that the initial saccadic end position (i.e., the position of the hypometric or hypermetric saccade) was not included in the final BCEA analysis.

### Inter-eye GH fixation BCEA comparison to assess conjugacy

There were no inter-eye differences in the GH fixation BCEA in control participants in all four trial directions and no inter-eye differences in MG patients in all four trial directions. Table 3 lists GH fixation BCEA means and SD values.

**Table 3:** GH fixation BCEA (mean and standard deviation) of control and MG right and left eyes.

GH direction	Eye	Control fixation BCEA (degree^2^)^*^ (*N* = 20)	MG fixation BCEA (degree^2^)^*^ (*N* = 20)
Left	R	1.74 ± 1.52	2.80 ± 2.64
	L	1.80 ± 1.10	2.81 ± 2.57
Right	R	1.82 ± 1.67	2.61 ± 2.56
	L	1.83 ± 1.67	2.47 ± 2.32
Up	R	1.80 ± 1.87	3.22 ± 3.27
	L	2.11 ± 2.24	3.10 ± 2.74
Downward	R	2.33 ± 2.20	3.40 ± 2.25
	L	2.38 ± 2.81	3.59 ± 3.10

* Values expressed as mean and standard deviation.

BCEA, bivariate contour ellipse area; GH, gaze hold; L, left; MG, myasthenia gravis; R, right.

A subgroup age group analysis in MG patients aged <50 and >50 did not reveal any inter-eye differences in the BCEA during a fixation task in any of the four directions. There were also no differences in the BCEA between the age groups (<50 vs. >50 years) of each eye. An analysis of X-axis SD and Y-axis SD did not demonstrate any MG inte-eye differences during a horizontal GH (left) and a vertical GH (upward).

### Inter-group GH fixation BCEA: control vs. MG

**Figure 3:**
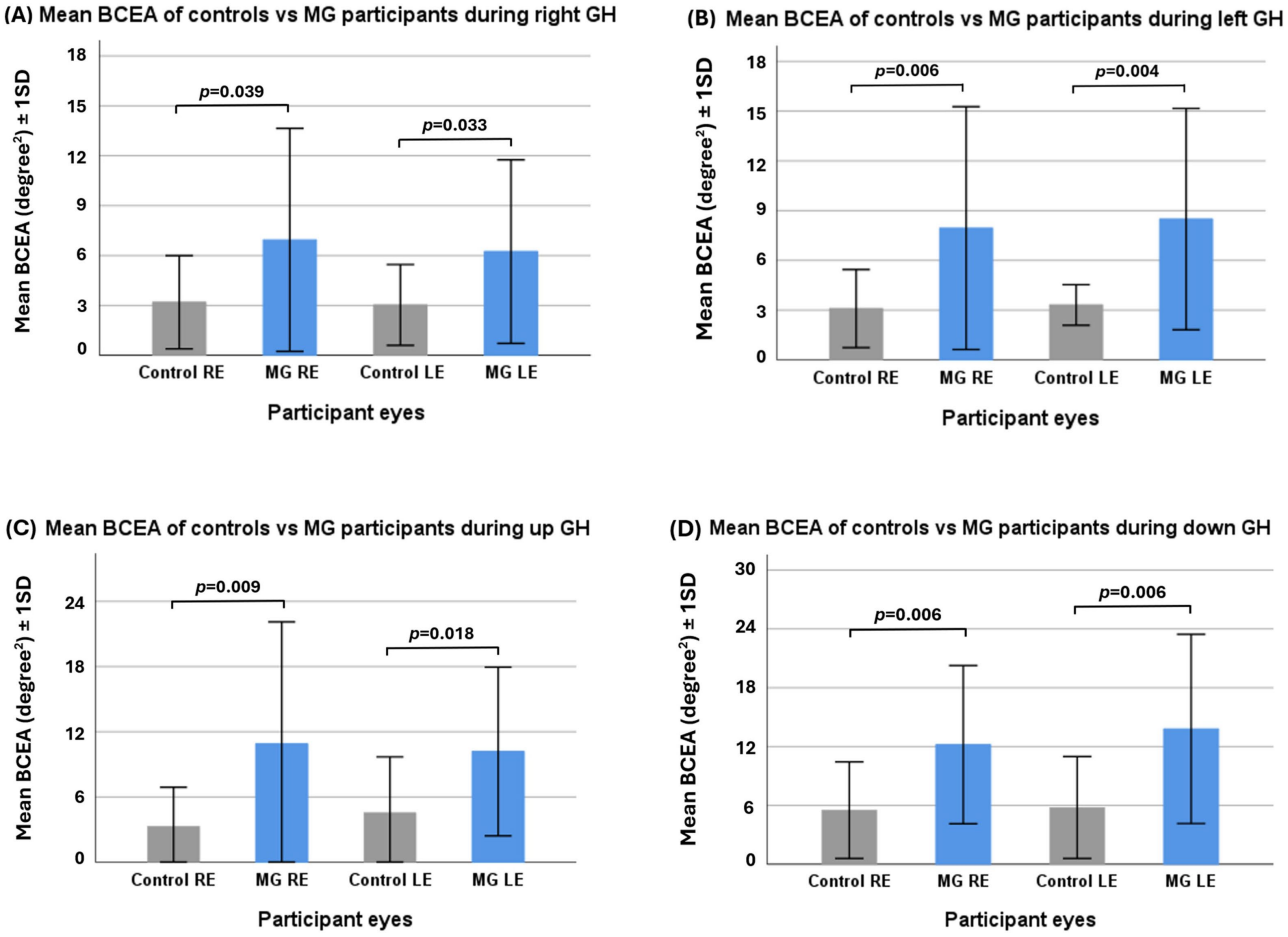
Independent samples *t*-test of control vs. myasthenia gravis (MG) participants’ mean fixation bivariate contour ellipse area (BCEA) in right gaze hold **(A)**, left gaze hold **(B)**, upward gaze hold **(C)**, and downward gaze hold **(D)**. GH—gaze hold.

In all directions of GH, MG patients exhibited a significantly greater BCEA in both eyes than control participants (see Figure 3). The BCEA of vertical gaze holds in MG patients was greater than that of horizontal gaze holds.

### Comparison of X-axis and Y-axis standard deviations in one horizontal and one vertical GH

An independent samples *t*-test using only the right eye in the left GH demonstrated a greater mean X-axis standard deviation (SD) in MG patients (0.43 ± 0.36 deg) than in control participants (0.44 ± 0.21 deg), *t*(37) = −2.18, *p* = 0.008. Similarly, the mean Y-axis SD was greater in MG patients (0.61 ± 0.01 deg) than in control participants (0.39 ± 0.20 deg), *t*(37) = −2.39, *p* = 0.022.

For the upward gaze GH, MG patients did not show any significant differences in X-axis SD compared to control participants, but they had a greater Y-axis SD (0.88 ± 0.70 deg) than control participants (0.38 ± 0.13 deg), *t*(36) = −3.03, *p* = 0.004.

MG patients with diplopia showed no significant differences in standard deviation compared to MG patients without diplopia.

### Inter-eye comparison of the number of GH refixations on the target

In each of the four gaze hold directions, there were no differences in the number of refixations between the eyes of control participants or between the eyes of MG patients.

### Inter-group comparison of the number of GH refixations on the target

**Figure 4:**
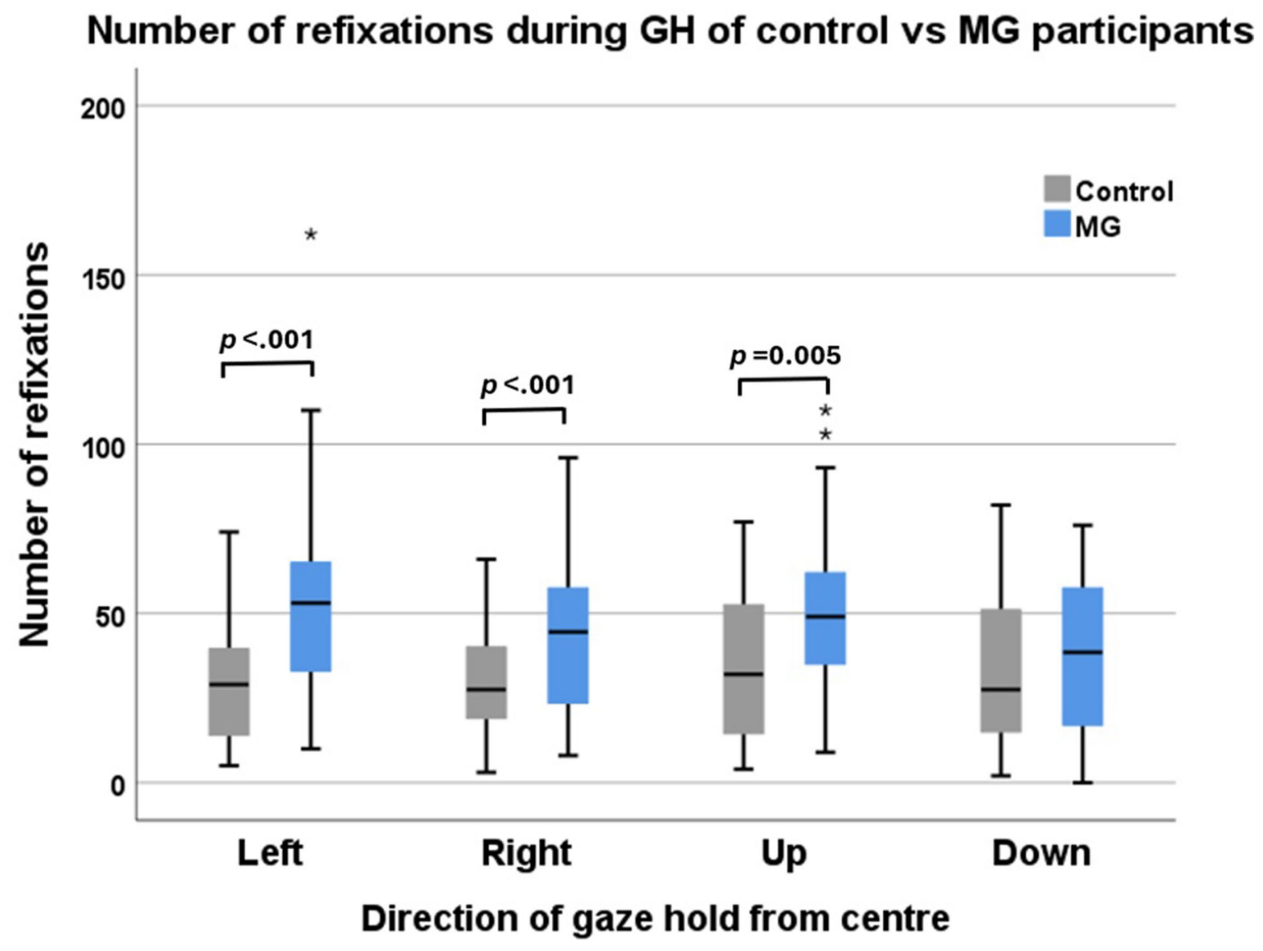
Number of refixations of control participants (grey) vs. MG patients (blue) during four directions of target gaze hold. The left and right eyes were combined for this analysis.

MG patients demonstrated a larger number of refixations in the left, right, and upward directions than did the control participants (Figure 4) but did not show a significant difference in the downward direction, although the mean was greater.

### Inter-eye comparison of target IA percentage of dwell time (conjugacy and accuracy)

In each of the four gaze hold positions, there were no differences in the target IA percentage of dwell time for consistency between the eyes of control participants or between the eyes of MG patients.

### Inter-group comparison of target IA percentage of dwell time (accuracy)

**Figure 5:**
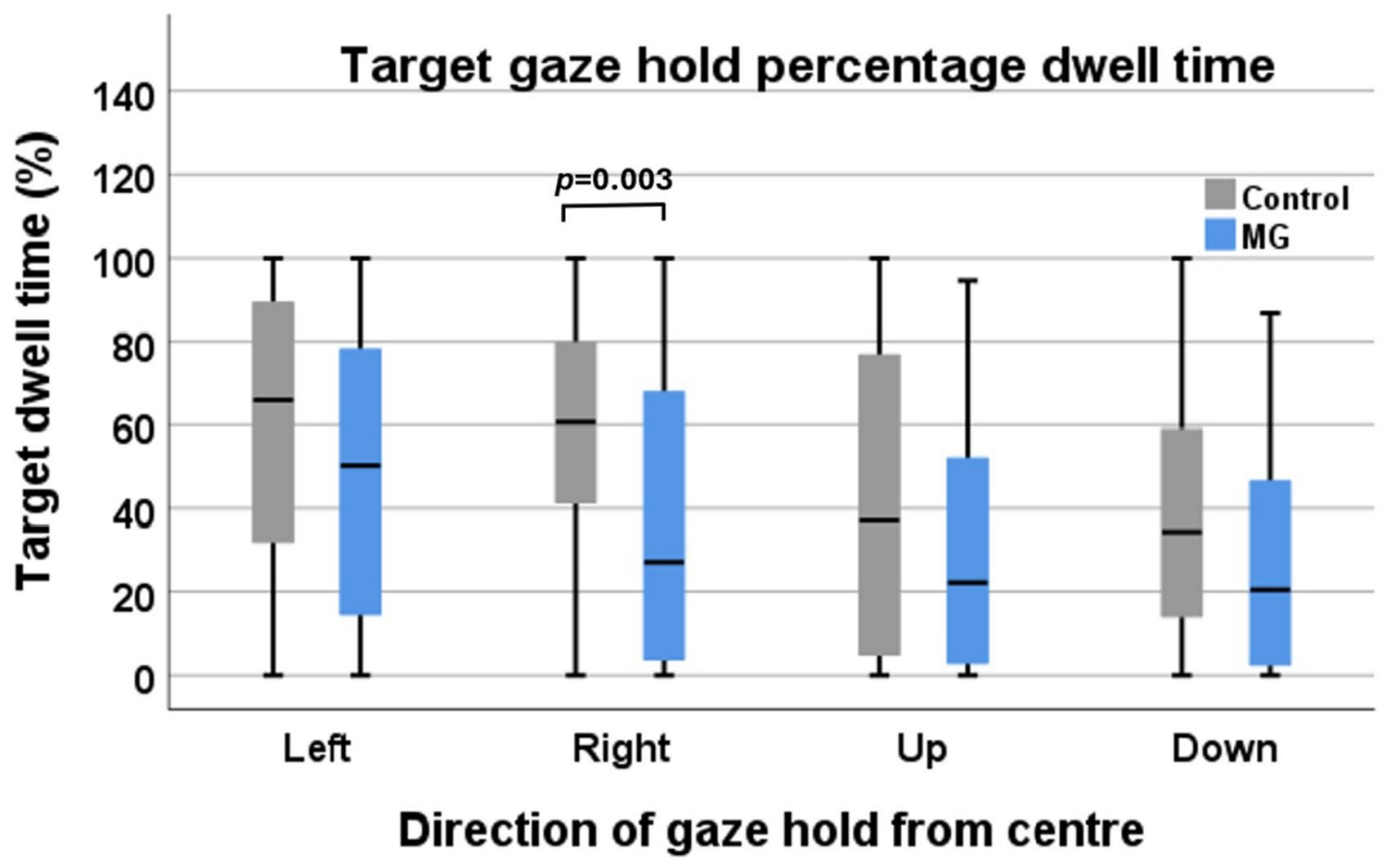
Target interest area percentage of dwell time of control participants (grey) vs. MG patients (blue) during four directions of target gaze hold.

With right and left eyes combined, the intergroup comparison only showed a significant difference in the right gaze hold. This was also the case when comparing the right and left eyes of controls vs. MG separately. However, the overall mean for the other gaze hold directions was lower than in the control groups. The vertical GH in both the control and MG groups was lower than the horizontal GH (Figure 5).

## Discussion

This study aimed to use GH fixation BCEA analysis to differentiate between healthy control participants and MG patients, adding to the current literature. There are currently very few studies looking at fixation instability using VOG (8, 9) as a marker of neuromuscular transmission failure in MG patients.

Visual fixation is defined as the maintenance of gaze on a certain point to keep the image on the fovea and is influenced by factors such as visual acuity, stereopsis, colour vision, and oculomotor control (10, 11). Visual fixation plays an important role in saccadic and pursuit eye movements. It also changes with age, with worse fixation stability early on in life during development and after the fifth decade, due to the development of ophthalmic or neurological conditions (12). Fixational eye movements have been classified into intersaccadic movements and microsaccades (10). Intersaccadic movements describe the movement of the eyes after a saccade (ocular drift and tremor) and microsaccades (also known as miniature, jerks, flicks, and fixational saccades); they are episodic events that occur approximately 1–2 times/s and that can be up to 2 deg in amplitude, with a higher velocity than drift movements (13). Microsaccades usually are considered to be binocular horizontal and vertical movements. Oblique microsaccades are less commonly observed. Small movements of the head, or blinking, even if compensated by rotatory eye movements can lead to the displacement of the retinal image.

In healthy individuals, there is an inherent instability of the oculomotor system that causes ocular drift of the image on the retina during fixation tasks (14). Normal fixation on a target has been proposed to require a degree of ocular drift (small amplitude, slow movements) to prevent perceptual fading of the image on the retina, and these are usually accompanied by microsaccadic corrections that may or may not be triggered to correct the drift itself (14–16). These microsaccadic movements can correct drift but may also produce errors in fixation.

Abnormal fixation presents as excessive microsaccades, saccadic intrusions, square-wave jerks, and nystagmus (17, 18). These may be representative of abnormalities in the oculomotor pathway more centrally in oculomotor control or peripherally in the extraocular muscles.

Central control of fixation involves a sustained firing rate of omnipause neurons located in the nucleus raphe interpositus of the paramedian pontine reticular formation. This tonic activity inhibits the firing of the premotor burst neurons responsible for saccadic eye movements located in the pontomedullary and mesencephalic reticular formation and maintains fixation at the end of the saccade (19). Without this tonic activity, after a saccade, the eyes would move back to the primary position due to the elastic properties of the antagonist EOMs and surrounding tissues (11). Previous studies have shown that MG patients demonstrate wavering fixation and backdrift at the end of saccadic eye movements during eye-tracking tests (20–22).

### Conjugacy of fixation in controls and MG patients

There were no inter-eye differences in GH fixation BCEAs, Y-axis SD, or y-axis SD in the control group, indicating that eye movements remain conjugate while maintaining fixation. This was unsurprising, as some authors have found that drift movements during fixation are, in general, binocularly synchronous (23). The results of the present study are consistent with this finding. Another study also found that Y-axis components of movement are significantly more synchronised than X-axis components between the eyes, and the authors hypothesised that this may be due to the greater need for movements in the horizontal plane to view stereoscopic images at different distances (24). This study also found that there was no synchronisation between X-axis and Y-axis components. Historically, Hering proposed the law of equal innervation between the eyes, while more recently it has been found that Hemholtz’s argument that each eye is under uniocular control is more representative of binocular eye movements (25), particularly when changing the depth of focus for near and far objects. In the case of a prolonged fixation task at a fixed distance, it is unlikely that we would see much disparity between the eyes during the task, apart from the initial movement to the target.

Interestingly, despite the suspected variable fluctuations in extraocular neurotransmission and variable extraocular muscle (EOM) involvement (26), the MG group showed no inter-eye differences in BCEA, X-axis SD, Y-axis SD, or target IA percentage of dwell time. No differences were detected in the BCEA analysis of the MG subgroups of individuals <50 years of age and >50 years of age. There are currently no studies looking at inter-eye differences in the BCEA of MG patients. Previous oculomotor studies have used the dominant eye only or have looked at MG eyes sequentially, but not simultaneously (8). The lack of inter-eye differences in the MG group may be due to changes in central gain to compensate for weak muscles in an attempt to maintain conjugacy and singular vision, or it is possible that the power of this study was too low to detect differences.

### Fixation instability of control participants vs. MG patients

There was more fixation instability, as measured by greater BCEA, in MG patients compared to controls in all directions of gaze hold. MG patients also had greater BCEA during vertical gaze holds compared to horizontal gaze, greater mean X-and Y-axis SD during left gaze, and greater mean Y-axis SD during upward gaze. The latter is consistent with a previous study (8). A HESS chart analysis of MG eyes has also found that the majority of ocular deviations occur during horizontal and upward gaze movements (27). Downward gaze was not previously known to lead to significant deviations, but this study showed that fixation instability occurred similarly in downward gaze.

Weakness of the elevators of the eye (superior rectus and inferior oblique) has been found to be more common in both controls and MG patients, possibly due to anatomical factors such as greater muscle bulk or fewer muscle spindles, as reported for the superior recti, and in addition, the physiological need for upward gaze is less required (26). Although both control and MG participants exhibited this finding, MG patients were found to be more significantly affected. This may explain the larger BCEAs during vertical gaze holds in MG patients compared to horizontal gaze holds and when compared to controls. It is postulated that the resting eye position is at or below the horizontal midline, which increases the force of contraction needed to elevate the eyes and therefore makes them more prone to fatigue in MG patients.

The number of refixations on and around the eccentric target was higher in the MG groups than in the controls in all GH directions, but there was no significant difference between the horizontal and vertical directions. This may be a sign of extraocular muscle fatigue. The extraocular muscles have different fibre types, which have been suggested to serve different functional roles. These include singly innervated fibres (SIF) and multiply innervated fibres (MIF) (28). SIFs comprise 80% of muscle fibres innervated by large-diameter myelinated axons. They are striated, fatiguable, and responsible for ballistic eye movements such as eccentric saccades. MIFs make up the rest of the muscle fibres, innervated by smaller and less myelinated axons, which produce a steady tonic contraction. MIFs are fatigue-resistant and play an important role in gaze hold (29, 30). If MIFS are fatigue-resistant, why is there more fixation instability in MG patients? EOMs, compared to skeletal muscle have been found to have a reduced neurotransmission safety factor. This means that they are more prone to NMJ instability from antibody receptor blockade due to reduced sarcolemmal folding and thus reduced concentration of nicotinic Ach-R at the post-synaptic junction (31). EOMs are also more prone to NMJ destruction from complement-mediated membrane attack complexes, due to downregulation of complement regulatory proteins such as decay accelerating factor (DAF) and upregulation of complement activators (32). Thus, in MG patients compared to control participants, their EOMs would be more prone to NMJ instability, which may present as fixation instability in this study.

In addition, the target IA percentage of dwell time was lower in MG patients than in controls. This differs from the BCEA as the BCEA is primarily a measure of the spread of refixations over time, whereas the percentage of dwell time explores the ability of the eyes to stay on target. It is possible that drifting off the interest area may be a sign of EOM fatigue. It was noted that control participants also demonstrated reduced dwell time. Fixation stability also depends on central processes. Participants may have been distracted or tired during the gaze hold test, impairing their ability to stay on target. There are currently no other studies that have looked on the target IA percentage of dwell time, and thus, this remains an area to explore.

## Limitations

This study is limited by the small number of participants and thus does not represent the entire population, despite including a heterogeneous group of ethnicities. This study tested more MG patients than the included 20, but these patients were excluded due to various factors such as severe fatigue and severe ptosis (despite taping the eyelid), which impacted their ability to complete the test adequately. Thus, patient factors may impact VOG as a diagnostic measure.

Patients had varying severities of MG and were on different treatments, which may impact the interpretation of results. Ideally, future studies with a larger patient sample and greater analytical power may show additional differences, such as inter-eye differences that were not apparent in this study.

## Conclusion

To the best of our knowledge, this is the first study to use BCEA analysis across multiple gaze directions in MG patients. It supports the existing literature, while further expanding the evidence of disparities in downward and horizontal gaze. Additionally, it found a higher number of refixations and a lower target IA percentage of dwell time in MG patients during GH. Taken together these findings are suggestive of fixation instability as a result of intermittent EOM weakness. Interestingly, there were no inter-eye differences in either the control or MG groups, the latter of which remains unexplained. This study only compared MG to control participants and no other ocular mimics. Further studies in the future are suggested to compare MG with other ocular mimics to determine whether the BCEA can be used clinically as a diagnostic tool using video-oculography. In conclusion, VOG analysis of BCEA may be helpful in detecting EOM weakness in cases where clinical signs at the bedside are not easily observed, particularly in patients with intermittent symptoms, such as those without obvious ptosis or ophthalmoplegia.

## Data Availability

The datasets presented in this article are not readily available because of data sensitivity and are available from the corresponding author upon reasonable request. Data are located in controlled access data storage at Monash University, Alfred Centre campus. Requests to access the datasets should be directed to mnnguyen02@gmail.com.
